# Vector-borne disease surveillance and control resource needs in Colorado public health organizations

**DOI:** 10.1371/journal.pone.0347142

**Published:** 2026-04-20

**Authors:** Jessica L. Butler, Jordan A. Crawford, Kayleigh P. Keller, Thomas Jaenisch, Molly M. Lamb, Juliana G. Barnard, Talia M. Quandelacy

**Affiliations:** 1 Department of Epidemiology, Colorado School of Public Health, Aurora, Colorado, United States of America; 2 University of Colorado, Denver, Colorado, United States of America; 3 Department of Statistics, Colorado State University, Fort Collins, Colorado, United States of America; 4 Center for Global Health, Colorado School of Public Health, Aurora, Colorado, United States of America; 5 Department of Pediatrics, University of Colorado, Aurora, Colorado, United States of America; Instituto Nacional de Salud Publica Centro de Investigaciones sobre Enfermedades Infecciosas, MEXICO

## Abstract

Since 2002, Colorado has experienced a high vector-borne disease (VBD) burden from diseases like West Nile fever, plague, and tularemia. Recent evidence underscores a lack of vector-borne surveillance and control program capacity across public health organizations, prompting our evaluation of funding and resource needs for such programs in Colorado. We assessed the needs, organizational capacity, and mitigation strategies of Colorado public health organizations’ VBD programs using a convergent mixed methods study design. Between January and May 2024, we surveyed and interviewed key public health organizations leaders. Findings were determined through mixed methods integration of survey data, thematic analysis on interviews, and content analysis on financial and policy documents. Overall, 28 public health practitioners (35% response rate) at 24 Colorado public health organizations (63% urban, 25% rural, 13% frontier counties) were surveyed and 18 were interviewed. We found only 37.5% of Colorado’s public health organizations conducted vector surveillance for any vector species. Smaller rural and frontier communities (<100K people) were particularly vulnerable to inadequate VBD organizational capacity and resource availability, and public health organizations in areas with populations < 10K or between 51-100K did not conduct vector surveillance. Interviewees perceived their VBD surveillance and program capacity as inadequate and reported significant limitations in their ability to carry out vector control activities. Moreover, they reported that competing priorities for emerging diseases resulted in reactionary rather than proactive responses to VBDs, highlighting the lack of sustainable funding for vector programs. As VBDs continue to increase in Colorado, outbreaks of diseases like West Nile fever or plague will likely go undetected without improved surveillance. The findings from our study have implications for where surveillance and control are lacking and highlight opportunities to continue improving and supporting surveillance and controls programs for VBDs.

## Introduction

Vector-borne diseases (VBDs) pose a significant public health threat globally [1]. They are a growing concern in the United States (US), where reported cases of VBD have more than doubled between 2001–2023 [2,3]. Colorado has experienced a high VBD burden since 2002 from diseases like West Nile fever [4,5], plague [6,7], and tularemia [8–10] and is currently experiencing increased incidence across the three aforementioned VBDs [5,6,9]. West Nile virus (WNV) is spread primarily through mosquitoes [11], while plague and tularemia are bacterial diseases of rodents and rabbits generally transmitted to humans through the bite of infected fleas or deer flies and ticks, respectively [12,13]. Tackling these issues is difficult given complex ecological factors. Transmission of vector-borne pathogens in Colorado occurs partly due to suitable local ecology and prevalence of capable vector species [4,14]. WNV carrying mosquitoes and most human cases are concentrated in populated lower-elevation areas along the eastern plains and Front Range in Colorado, where irrigation practices create ideal mosquito habitats (ditches, marshes) especially during drier summer conditions [5,15–18]. Tick-borne disease risk (e.g., Colorado tick fever virus) increases with elevation and human cases cluster in mountain towns and recreational outdoor zones that contain grass, sagebrush, or conifer and aspen trees. [19–21]. Human-vector exposures often occur through outdoor occupations and hobbies (e.g., agricultural workers, hiking, hunting) [22]. A 2017 report estimated 92% of Colorado residents participate in outdoor recreation each year [23], placing them at heightened risk for exposure to vectors. Older adults and immunocompromised individuals are also at increased risk of severe disease from WNV [24], while tularemia and plague can be life-threatening even with early diagnosis and antibiotic treatment [25,26].

VBD surveillance programs monitor changes in both human and vector populations, and are critical to preventing and responding to outbreaks. Human surveillance and control focus on reported syndromic cases, active case investigation, and outbreak response [27]. Vector surveillance uses standardized trapping and speciation of mosquitoes, fleas and ticks, and vector control reduces vector populations through biological and chemical products, environmental management, and pesticide resistance testing [28,29]. Together, these efforts form the foundational vector program competencies aimed at reducing VBD transmission risk.

Despite their importance, VBD surveillance and control programs are often overburdened and under-resourced [2]. A 2018 Morbidity and Mortality Weekly Report (MMWR) found persistent, locality-specific risks and a rising threat from emerging VBDs (e.g., Lyme disease and WNV), which have increasingly encumbered local and state health departments [2]. Similarly, recent reports highlight the increased strain on local and state public health agencies in addressing increased VBD threats. In 2017, a national assessment of vector control program competencies found that 84% of 1,083 local mosquito control organizations reported lacking one or more of five core vector control competencies [30]. A 2020 follow-up national assessment [31] found vector control programs in Region 8 (Colorado, Montana, North Dakota, South Dakota, Utah and Wyoming) were less likely to be fully capable compared to other regions, with 87% of the programs in Region 8 characterized as ‘needs improvement’ and only 13% noted as ‘fully capable’ [28]. In the most recent 2023 assessment, based on 192 programs, capacity for nearly all mosquito surveillance and control activities dropped slightly from 2020 to 2023, falling between 1% and 11% [28]. These surveys highlight the need for improvement of vector control programs. Building sustainable VBD surveillance and control programs with access to sensitive and specific testing, accurate diagnostic tools, real-time reporting and rapid implementation of control techniques remains challenging [2].

Vector control programs in the United States are nearly all locally funded and operated, but little data exist on what resources are available to support these programs. Maintaining and improving surveillance systems is foundational to public health prevention of disease, as is evaluating processes and resources needed to support these functions. Given the significant burden of VBD and increased cases in recent years in Colorado and limited knowledge of current resources and capacity for VBD programs, we studied the needs of VBD programs and explored the organizational capacity and mitigation strategies for VBDs by Colorado state and local public health organizations using a mixed methods study design. We interviewed and surveyed key public health practitioners from public health organizations between January and May 2024.

## Materials and methods

### Study design

**Fig 1:**
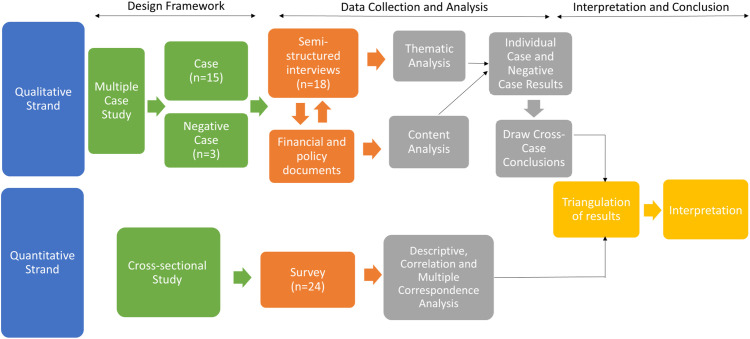
Case Study Convergent Mixed Methods Study Design. There were two strands of this study design: the qualitative and quantitative strands (blue). The qualitative strand of our study design included a multiple case study with 15 cases and three negative cases (green). Data were collected by conducting 18 semi-structured interviews and gathering relevant financial and policy documents (orange). Interviews and documents were reviewed in tandem to enhance our understanding of the individual cases (orange). Semi-structured interviews underwent thematic analysis, and documents had their content analyzed (gray). Individual case and negative case results informed cross-case conclusions (gray). The quantitative strand used a cross-sectional survey of 24 public health practitioners (orange). Data were analyzed through descriptive, correlation and multiple correspondence analysis (gray). Finally, results from the qualitative and quantitative strands were triangulated, integrated, and interpreted (yellow).

We used a convergent mixed methods study design [32] with a case study methodology [33] to enable triangulation of quantitative and qualitative methods and data (Fig 1). The quantitative strand employed a cross-sectional survey. The qualitative strand relied on a collective case study methodology multi-case design [33] with semi-structured interviews, given that the vector-borne surveillance and control program’s performance is highly tied to the local context. An important consideration was how the program operated at the state, county and city department level, necessitating a multi-case design. We compared case and negative case organizations to strengthen the validity and robustness of our findings – negative cases help to refine or even rethink initial assumptions and lead to a more nuanced and accurate explanation of the emerging patterns observed. A case was defined as a state or local public health organizations that conducted vector-borne disease surveillance and/or control activities. A negative case was defined as a health department located in an area where the burden of vector-borne disease is low and where routine vector-borne disease surveillance and control activities are not conducted. Pathogen ecology, vector taxa, and reservoir host activity questions were outside the scope of our research. While we did seek to understand differences between rural and urban public health organization settings, our study did not focus on the ecology of these different regions.

#### Recruitment.

Eligible study participants were current public health practitioners employed by Colorado health departments with direct VBD surveillance responsibilities including collection, management, analysis, or response (e.g., public health leaders, data analysts, epidemiologists, environmental health specialists, public health nurses and administrators). We used a non-probability convenience sampling approach to recruit survey participants and a purposeful sampling strategy [34] to select participants for the semi-structured interviews. Participants were recruited for balanced representation of urban, rural and frontier public health departments [35].

Public health practitioners were recruited in three ways: 1) outreach via e-mail to a Colorado communicable disease network; 2) promotion via monthly call to public health employees; 3) outreach via e-mail by the National Association of City and County Health Officials (NACCHO). Respondents were invited to participate in the electronic survey and upon completion were invited to participate in a 45-minute semi-structured interview. Two follow-up reminder emails were sent to respondents. Every effort was made to schedule interviews within two to three weeks of survey completion.

#### Exclusion criteria.

We excluded individuals from our survey analysis if they did not provide consent to participate in the survey or if they failed to answer all questions. We excluded participants from participating in the semi-structured interviews if they did not include their name in the survey.

#### Ethics statement.

Participants were informed of the voluntary nature of this research and provided written consent in the study questionnaire and those invited to be interviewed provided oral consent before beginning the interview which was witnessed by the interviewer and documented via voice recording by the interviewer. This study was approved by the Colorado Multiple Institutional Review Board (COMIRB) (23–1754).

### Data collection

#### Questionnaire administration.

We developed a 53-question survey adapted from the World Health Organization framework [36]. The survey took 10–20 minutes to complete and included close-ended (e.g., yes/no, multiple choice) and free-response questions to assess public health organizations organizational capacity and resources related to vector-borne surveillance and control activities, and climate risk and vulnerability (S1 Appendix: Survey).

#### Semi-structured interviews.

An interview guide was developed from the Vanderbilt University Medical Center Community Health Needs Assessment framework [37] and reviewed by infectious disease public health and qualitative method experts. Interview questions were comprised of structured and open-ended questions exploring how participants engaged vector-borne related activities and barriers experienced (S2 Appendix: Interview Guide). Participants were interviewed for approximately 45 minutes by two interviewers. There were no repeat interviews. Insights gained informed subsequent interviews [38]. The interviews were conducted via video conference, dual recorded, and transcribed subsequently using Trint transcription software [39] and edited by the interviewers until they were verbatim. Handwritten field notes were transcribed and included in the thematic analysis. Team-based coding was employed to reach consensus on codes and review emerging themes.

### Analysis framework

#### Survey analysis.

We evaluated responses to survey questions about public health organization surveillance and response capacity to vector-borne disease threats. In cases where multiple individuals from the same health department responded to the survey (n = 3), an individual was selected at random so that each health department was represented an equal number of times in the survey data for analysis. We excluded from the analysis: responses to 16 questions due to low response counts and questions with less than 5 responses, which primarily occurred due to branching logic of the survey.

Counts (n) and percentages (%) were computed for all multiple-choice questions related to vector-borne disease surveillance and control capacity within public health organizations. Responses related to climate risk and vulnerability are reported elsewhere. Responses to open-ended questions were reviewed and manually assigned to corresponding levels. Survey responses were stratified by populations served: < 10,000, 10,000–50,000; 51,000–100,000; > 100,000 to assess differences across public health organizations that service populations reflective of frontier (population density of six or fewer persons per square mile), rural (non-metropolitan counties without cities of 50,000 or more residents) and urban (population of 50,000 or more, including large towns [51–100K] and cities [100K+]) areas [35]. We also evaluated responses from participants with missing name information to assess if those respondents were different from other respondents.

Supplemental analyses included multiple component analyses (MCA) to evaluate relationships among survey responses and determine if differences of survey responses by population size were present; and display 2023 rates of WNV among public health organization participants (S1 Text). Survey data were analyzed using R (version 4.1.2) [40].

#### Thematic analysis from interviews.

Thematic analysis was performed by two study team members (JB and JC) in an iterative manner to conceptualize the data, group concepts into patterns and relationships, and analyze across cases for similarities and differences. Results are based on case experiences and feedback, and were identified by comparing the case to negative-case sites. Data were analyzed using both inductive and deductive coding. The study team members read through the interview records to become familiar with their content before coding. A deductive codebook was developed based on the interview guide. Inductive codes were added to the codebook as identified during coding. Twenty percent of the interviews were dual coded, and the coding team worked to find consensus on codes and code definitions to establish coding consistency. Team meetings were held where codes were compared and discrepancies addressed and reconciled, and additional study team members (JGB and TMQ) provided content and methodological input. Memos were created to capture preliminary ideas about potential themes and describe illustrative cases. Themes were finalized through a process of running queries of the data, and comparing and reconciling emerging ideas into preliminary themes until a point of saturation was reached. Documents were read, coded and linked to the interview data to triangulate information from multiple sources, participants, and points in time. Atlas.ti (version 24.1.1) software was used for all thematic coding and analysis [41].

### Integration procedures

We separately analyzed quantitative and qualitative data, then compared their findings related to public health organization vector-borne disease surveillance and control capacity. We compared categories, patterns, and variables from survey results to themes emerging from the interviews. The data were interpreted based on how the confirming or disconfirming evidence from the merged results enhanced understanding of the research problem. We reported the quantitative and qualitative results in a side-by-side strategy [32]. We identified the final study results by considering convergence, expansion, complementarity, or discordance of the qualitative and quantitative findings. Assertions and conclusions were summarized to inform our understanding of the cases. Suggestions were proposed to prioritize the needs of the Colorado vector-borne divisions, identify potential solutions to resources concerns and make recommendations for successful prevention and containment strategies, given the resources available.

## Results

**Fig 2:**
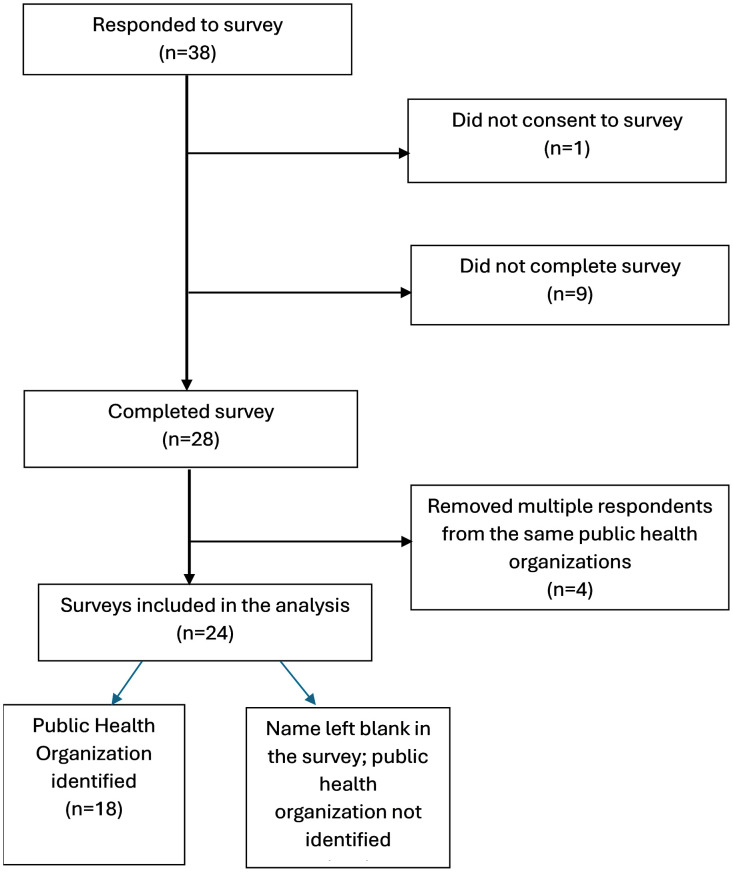
Study Inclusion and Exclusion Schematic. Of the 38 individuals who responded to invitations to participate, one participant did not consent to the study and nine did not complete the survey leaving 28 completed surveys. Four individuals who responded from the same public health organizations were removed. 24 public health organizations were represented in our analysis, with six being unable to be identified given respondents left their name blank.

Data were collected from January 24^th^ to May 17^th^, 2024, from 24 public health organizations. The survey was distributed to 81 public health practitioners, yielding a 35% response rate (28 completed responses). In total, 38 individuals started the survey (Fig 2). Of these, 10 individuals did not complete the survey: one individual did not consent to participate and nine others did not complete the survey. This resulted in 28 individuals who fully completed the survey. These 28 respondents represented 24 unique public health organizations. We excluded four individuals who responded from the same public health organization. Among the 28 survey completers, six did not provide identifying information and were thus ineligible for follow-up interviews (21%), although their data was retained for quantitative analyses. Nineteen of the remaining 22 individuals were invited to participate in semi-structured interviews, and 18 agreed to participate (15 case participants and three negative cases), resulting in a 94.7% interview response rate. The remaining three individuals were not invited for interviews because thematic saturation had already been reached.

### Sample characteristics

Survey respondent characteristics are presented in Table 1. Respondents included Epidemiologists (42%), Environmental Health Specialists (33%), Administrators (13%) and Public Health Nurses (13%). Most reported having a Master’s degree (63%) and 42% reported at least 10 years of experience. Most respondents were from county health departments (83%). Geographically, 63% were in an urban setting, 25% in a rural setting and 13% in a frontier setting. Respondents from unidentified public health organizations largely held a role in Environmental Health (67%) but otherwise shared similar characteristics as the overall sample.

**Table 1:** Characteristics of Colorado Public Health Organization Respondents Surveyed from January to May 2024.

	Public Health Organizations Serviced Population Size^1^					
**Characteristics**	**Overall** **(N = 24)**	**<10K** **(N = 2)**	**10-50K** **(N = 4)**	**51-100K** **(N = 4)**	**100K +** **(N = 8)**	**Public Health Organization Unknown**^**2**^ **(N = 6)**
**Job Role**						
Administrator	3 (13%)	1 (50%)	1 (25%)	0 (0%)	0 (0%)	1 (17%)
Public Health Nurse	3 (13%)	1 (50%)	2 (50%)	0 (0%)	0 (0%)	0 (0%)
Epidemiologist	10 (42%)	0 (0%)	1 (25%)	3 (75%)	5 (63%)	1 (17%)
Environmental Health	8 (33%)	0 (0%)	0 (0%)	1 (25%)	3 (38%)	4 (67%)
**Education**						
High school diploma or equivalent	1 (4%)	0 (0%)	0 (0%)	1 (25%)	0 (0%)	0 (0%)
Bachelor’s degree	6 (25%)	0 (0%)	1 (25%)	0 (0%)	3 (38%)	2 (33%)
Master’s degree	15 (63%)	2 (100%)	3 (75%)	3 (75%)	3 (38%)	4 (67%)
Doctoral or professional degree (MD, DO, PharmD, etc.)	2 (8%)	0 (0%)	0 (0%)	0 (0%)	2 (25%)	0 (0%)
**Years of Experience**						
0-5 years	9 (38%)	0 (0%)	2 (50%)	0 (0%)	5 (63%)	2 (33%)
6-10 years	5 (21%)	1 (50%)	1 (25%)	1 (25%)	0 (0%)	2 (33%)
10 + years	10 (42%)	1 (50%)	1 (25%)	3 (75%)	3 (38%)	2 (33%)
**Administrative Region**						
County Health Department	20 (83%)	2 (100%)	4 (100%)	3 (75%)	6 (75%)	5 (83%)
Other	4 (17%)	0 (0%)	0 (0%)	1 (25%)	2 (25%)	1 (17%)
**County Type** ^**3**^						
Urban	15 (63%)	0 (0%)	1 (25%)	2 (50%)	8 (100%)	4 (67%)
Rural	6 (25%)	0 (0%)	3 (75%)	2 (50%)	0 (0%)	1 (17%)
Frontier	3 (13%)	2 (100%)	0 (0%)	0 (0%)	0 (0%)	1 (17%)

^1^Residential population size served by public health organization as reported by participants

^2^Represents public health organization where individuals left their name blank in the survey

Abbreviation: K = 1,000; MD = doctor of medicine; DO = doctor of osteopathic medicine; PharmD = doctor of pharmacy.

^3^Urban (population of 50,000 or more), rural (non-metropolitan counties without cities of 50,000 or more residents), frontier (population density of six or fewer persons per square mile).

**Fig 3:**
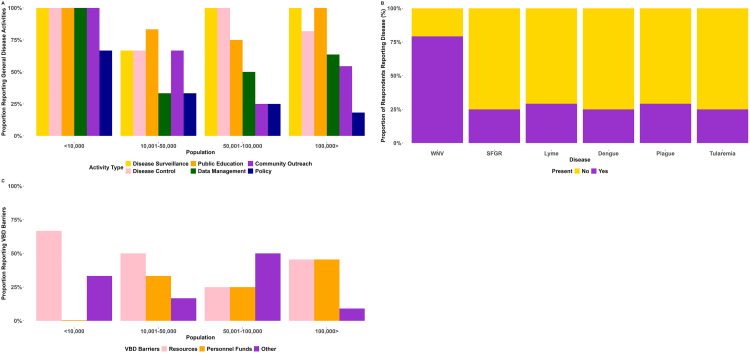
General Disease Prevention Efforts, Reported Vector-Borne Disease Activity and Barriers Among Colorado Public Health Organizations. Panel A shows the proportion of general disease activities reported by survey respondents and stratified by population size served by the public health organization. Panel B shows the vector-borne diseases reported by respondents over previous 12 months. Panel C shows the reported barriers to VBD prevention and control activities by population size. Colors reflect county population size serviced by public health organizations. The “Other” category reflects community buy in, political will, prioritization. Personal funds were not reported for locations with population size less than 10,000.

Respondents highlighted a number of activities conducted by public health organizations. Nearly all survey respondents reported activities related to general disease surveillance (92%) and control (83%), and public education (92%). Half of public health organizations reported data management (58%), community outreach (58%), and some policy (29%) activities (Fig 3A).

#### Reported VBDs and perceptions of VBD burden on priority setting.

The most common VBD reported in the past 12 months by survey was West Nile Virus (82%) followed by plague (32%), spotted fever group rickettsiae (SFGR) (28.6%) and tularemia (25.0%) (Fig 3B). Lyme disease (32%) and dengue virus (DENV) (29%) were also reported as travel acquired diseases (Fig 3B). The higher annual incidence rates of WNV observed across many counties was also highlighted as a concern by interviewees underscoring the importance of prioritizing VBDs. As one respondent told us *“… Colorado has typically and historically had pretty high cases of human West Nile virus. We just had a close to a record-breaking year the* *summer of 2023. We had over 600 human cases. And that’s a disease of, you know, not only that can cause death… it requires a lot of treatment, hospitalization, sometimes followed by extensive physical therapy afterwards.”* (Veterinary Epidemiologist, 100K+) The predominance of WNV in Colorado is also supported by other documents. For example, in 2023, Colorado experienced the worst WNV outbreak in the U.S. and one of the deadliest years for WNV since its arrival in the U.S. in 1999 [42] (Table 2). A health advisory on July 6, 2023 also noted WNV had been detected during routine mosquito surveillance in Boulder, Delta, Larimer and Weld counties (Table 2) [42]. In a press release disseminated in August of 2023, the state epidemiologist noted “the trends we are seeing in our West Nile virus tracking data are unprecedented” (Table 2) [43].

**Table 2:** Additional Data Sources for Contextualizing Cases.

Documents selected	Data analyzed
Health Advisory West Nile virus detected in Colorado mosquitoes | July 6, 2023 [42]	Recipients of Health Alert Network Broadcast
Communication: Colorado’s first human death of West Nile virus for 2023 [43]	State health officials seeing concerning trends of West Nile virus
Colorado’s Public Health Improvement Plan 2024 [44]	Goals and strategies related to vector-borne disease surveillance and control mechanisms
Centers for Disease Control and Prevention Public Health Emergency Preparedness Funding [45]	Historical funding
Centers for Disease Control and Prevention Fiscal Year 2023 Grants Summary Profile Report for Colorado [46]	Data for funding by department category and sub-category to determine vector-borne disease funding
Colorado Department of Public Health and Environment FY24 R-09 Maintain EpiTrax Disease Surveillance Platform [47]	Role of EpiTrax as a new disease surveillance modernization

Documentation was sought to triangulate evidence and further explore the cases via fiscal year budgets for public health funding; Public health organization’s technical reports; Colorado health advisories for vector-borne diseases in 2023, and Colorado Department of Public Health and Environment and National Vector-Borne Disease Strategic Plans.

In areas where VBD burden is consistently low, interviewees described uncertainty in whether there is justification for prioritizing resources devoted to VBDs, *“… you see programs like in Larimer County where they’re doing more surveillance. And we are, you know, lacking that. So do we have a problem? Don’t know, because we’re not looking for anything.”* (Administrator, 10-50K) The perceived burden of disease was a key factor in determining whether public health organizations prioritize VBD activities.

#### Vector surveillance and control organizational capacity and training needs.

Limited vector surveillance activities were reported in both surveys and interviews. Only nine public health organizations (38%) reported routine vector surveillance (defined as vector collection and/or vector pathogen testing) (Table 3). For the public health organizations conducting vector surveillance, 89% conducted vector collections, but only 11% engaged in pathogen testing, and half (56%) conducted activities during the summer. Survey responses showed that capacity for vector surveillance activities differed by population size (Table 3). Those conducting their own vector surveillance were often (63%) from larger populations (i.e., 100,000 residents or more).

**Table 3:** Vector Surveillance, Control and Resource Activities of 24 Colorado Public Health Organization Respondents Surveyed from January to May 2024.

	Public Health Organization Serviced Population Size					
	Overall (N = 24)	<10K (N = 2)	10-50K (N = 4)	51-100K (N = 4)	100K+ (N = 8)	Public Health Organization Missing^2^ (N = 6)
**Surveillance Activities**						
*Conduct VBD Surveillance*	9 (38%)	0 (0%)	2 (50%)	0 (0%)	5 (63%)	2 (33%)
*Surveillance Type*						
Vector collections	8 (89%)	0 (0%)	2 (50%)	0 (0%)	4 (50%)	2 (100%)
Pathogen testing	1 (11%)	0 (0%)	0 (0%)	0 (0%)	1 (13%)	0 (0%)
*Surveillance Period*						
All year	4 (44%)	0 (0%)	1 (25%)	0 (0%)	3 (38%)	0 (0%)
Summer	5 (56%)	0 (0%)	1 (25%)	0 (0%)	2 (25%)	2 (100%)
*Conduct GIS*	16 (67%)	1 (50%)	2 (50%)	3 (75%)	6 (75%)	4 (67%)
**Control Activities**						
*Control Activities Conducted for Vectors*						
Mosquitos	11 (46%)	0 (0%)	2 (50%)	1 (25%)	4 (50%)	4 (67%)
Bed bugs	1 (4%)	0 (0%)	1 (25%)	0 (0%)	0 (0%)	0 (0%)
None	12 (50%)	2 (100%)	1 (25%)	3 (75%)	4 (50%)	2 (33%)
*Conducts Insecticide Spraying*	4 (17%)	0 (0%)	0 (0%)	1 (25%)	2 (25%)	1 (17%)
*Insecticide Resistance Testing*						
No	21 (88%)	2 (100%)	4 (100%)	4 (100%)	5 (63%)	6 (100%)
Don’t know/unsure	3 (13%)	0 (0%)	0 (0%)	0 (0%)	3 (38%)	0 (0%)
**Resource Activities**						
*Reliable Testing Equipment*						
No	2 (8%)	0 (0%)	0 (0%)	0 (0%)	2 (25%)	0 (0%)
Yes	3 (13%)	0 (0%)	0 (0%)	1 (25%)	1 (13%)	1 (17%)
Not applicable	19 (79%)	2 (100%)	4 (100%)	3 (75%)	5 (63%)	5 (83%)
*Reliable Insecticide Application*						
Yes	4 (17%)	0 (0%)	1 (25%)	1 (25%)	1 (13%)	1 (17%)
Not applicable	20 (83%)	2 (100%)	3 (75%)	3 (75%)	7 (88%)	5 (83%)
*ID Samples Sent to Outside Testing Lab*	20 (83%)	2 (100%)	3 (75%)	3 (75%)	7 (88%)	5 (83%)
*Testing Methods Needed*						
Pathogen testing	4 (17%)	1 (50%)	1 (25%)	0 (0%)	2 (25%)	0 (0%)
Insecticide resistance testing	8 (33%)	0 (0%)	0 (0%)	1 (25%)	4 (50%)	3 (50%)
Don’t know/unsure	12 (50%)	1 (50%)	3 (75%)	3 (75%)	2 (25%)	3 (50%)
*Training Needed*						
Vector surveillance	10 (42%)	1 (50%)	3 (75%)	1 (25%)	4 (50%)	1 (17%)
Vector testing	2 (8%)	1 (50%)	1 (25%)	0 (0%)	0 (0%)	0 (0%)
Vector control	5 (21%)	0 (0%)	0 (0%)	1 (25%)	3 (38%)	1 (17%)
Insecticide resistance evaluations	1 (4%)	0 (0%)	0 (0%)	1 (25%)	0 (0%)	0 (0%)
Other^1^	4 (17%)	0 (0%)	0 (0%)	0 (0%)	1 (13%)	3 (50%)
Don’t know/unsure	2 (8%)	0 (0%)	0 (0%)	1 (25%)	0 (0%)	1 (17%)
*Optimal Method of Training*						
In Person, Onsite	11 (46%)	1 (50%)	2 (50%)	1 (25%)	5 (63%)	2 (33%)
Workshop, Offsite	2 (8%)	0 (0%)	1 (25%)	1 (25%)	0 (0%)	0 (0%)
Online Webinar	8 (33%)	0 (0%)	1 (25%)	2 (50%)	3 (38%)	2 (33%)
Other^2^	3 (13%)	1 (50%)	0 (0%)	0 (0%)	0 (0%)	2 (33%)

^1^emerging diseases, program management, funding allocation, all of the above.

^2^quality of training more important than modality, all of the above.

Interviewees highlighted the disparity in mosquito surveillance across Colorado, particularly underscoring the need for programs in rural and frontier counties, “*...I think the increased capacity for mosquito surveillance [is needed] for all parts of the county and not just, you know, the suburban ones, because I feel like that’s where a lot of, you know, effort is spent in the more populated areas. But that doesn’t* *mean that people aren’t living in the rural or frontier parts [of* *the] county that also need this sort of information or tools.”* (Infectious Disease Epidemiologist, 100K+)

Reported vector control capacity (defined as vector spraying or other vector population reduction activities, and insecticide resistance monitoring) was sparse across the public health organizations. While 46% of 24 public health organizations reported conducting vector control activities for mosquitoes (defined as chemical, biological, source reduction, or environmental management), only 17% of public health organizations performed insecticide spraying for any vector (Table 3). Of those who applied insecticides, 75% reported doing so weekly during the summer months and also were more likely to report conducting vector surveillance activities (50%). However, most respondents (88%) reported they did not conduct insecticide resistance testing, and 12% responded they do not know or were unsure.

Vector pathogen testing equipment and insecticide application resources were largely unavailable to public health organizations. Only 13% of respondents reported having reliable in-house testing equipment and 17% reported having up-to-date insecticide application equipment (Table 3). Most respondents (83%) also reported using outside laboratories for testing. When asked what additional testing methods practitioners would like to use in their organization, but do not currently have the resources for, 33% reported insecticide resistance testing, 17% pathogen testing, and 50% were unsure. Vector surveillance (42%) and vector control trainings (21%) were reported as the most important type of training needed in their organizations. In person, onsite trainings was the modality preferred by 46% of respondents.

#### Challenges to VBD mitigation efforts.

Resources (e.g., programmatic funding, technological tools, and trainings) were the most important barrier to VBD prevention and control reported by public health organizations (46%), followed by personnel funds (33%) and other (21%) (Fig 3C). The “other” category reflects community buy in, political will, prioritization. Interviews highlighted limits on public health organization capacity, specifically, noting no ability to carry out active mosquito surveillance or control activities within their public health organizations. *“Most jurisdictions in Colorado don’t have the capabilities to do any mosquito control, and they are relying solely on their residents protecting themselves.”* (Program Manager, 100K+)

The overwhelming majority of interviewees reported that mosquito control activities are contracted out to external organizations, which is consistent with only 46% of public health organizations who reported conducting control activities for mosquitoes in the survey. *“I think here [mosquito surveillance] it’s contracted out. I’m actually not certain exactly what we do. But I know that, like, nobody on our team is...going collecting mosquitoes or anything like that*.*”* (Senior Epidemiologist, 100K+) In the smallest (<10K) communities’ resources were of most concern (67%), while personnel funds and other barriers were more commonly reported among respondents from public health organizations serving communities with 10-100k residents (Fig 3C). Training as a barrier was only reported for population size 10-50k. Interviewees cited similar challenges, such as the time intensive nature of finding skilled public health practitioners and the extensive training required for the position. *“…I think our capacity is-- just our sometimes our lack of humans or lack of staff. If things get out of hand, we do jump in, you know?... but then you’re looking at just-in-time training.”* (Disease Control Specialist, 51-100K)

#### Lack of standardization and integration of vector-borne disease data.

Respondents reported data modernization, standardization and integration challenges with vector-borne disease data. They reported difficulty in managing surveillance systems, accessing historical case data and linking related vector data across different platforms, as described by this participant, *“So that’s one thing that we’ve been lacking. We’ve been housing our vector-borne data in a very outdated, really not very functional database.”* (Program Manager, 100K+)

Many respondents mentioned the upcoming transition to the EpiTrax system [a new core disease surveillance system that will consolidate the functions of 10 + existing State surveillance systems and modernize Colorado’s disease control infrastructure] may alleviate some of these concerns by allowing for visualizing disease trends and understanding real-time disease incidence. As one interviewee noted, *“...I think this is hopefully in the plans with the EpiTrax rollout…we have a separate like, the animal stuff lives in a separate database. So. It’s tricky to manage multiple surveillance systems. So, integrating all of those would be amazing.”* (Epidemiologist, 10-50K)

A Colorado Department of Public Health & Environment FY 2023–2024 Funding Request for the EpiTrax Disease Surveillance Platform [47] supported these sentiments noting, “the State’s systems are outdated, unreliable, and poorly supported” and “modernization of disease reporting infrastructure will prepare the Department for future pandemics and outbreaks as well as daily disease control and prevention efforts.” Improvements in surveillance systems can provide the data needed for geographic information system (GIS) analyses. 67% of public health organizations report having staff who perform GIS (Table 3), enabling more precise mapping of vector populations, identification of historical trends, and detection of high-risk areas.

#### Reactionary response to vector-borne outbreaks.

Interviews revealed many public health organizations have operated “more reactionary” in response to current and ongoing public health threats with less focus on prevention measures. As noted by one interviewee, “...*right now we’re not really all that active. We’re really reactive… it would probably be more useful if we were looking--like regularly looking--in areas that are likely places that those ticks might settle in Colorado, and we just don’t have the resources to do that.”* (Program Manager, 100K+)

Many respondents highlighted the need for more “proactive [surveillance] efforts” and underscored that their day-to-day activities involve mostly response efforts with little bandwidth for additional planning and preparation for future outbreaks, as described by one interviewee, *“…when I think of, our vector disease, we’;re able to do the day-to-day response, investigate the disease issues that come up. Outside of that, we’re not doing a ton of other sort of proactive planning efforts...”* (Program Coordinator, 100K+) Rather than engaging in prevention techniques consistently to proactively approach VBDs in their communities they often only increase efforts when an outbreak occurs.

#### Perceived lack of support and threats to sustainable funding.

Interviewees reported struggling to maintain sustainable funding for VBD programs that enhance prevention efforts and highlighted how difficult it is to convey disease mitigation successes. As noted by one interviewee, *“I think for, for most folks, just really trying to secure sustainable funding, for what we’re trying to do now, let alone being able to expand in the future and not have it just be reactionary. That is, unfortunately though the, the recipe I’ve seen throughout my career is we have to wait for things to get to a pretty catastrophic level before we start allocating funds for it. We don’t stay in the proactive prevention stage, but nobody can see those results. You don’t see all the things [local public health agencies] didn’t allow to happen. So that’s the challenge*.*”* (Program Coordinator, 100K+)

Interviewees reported difficulty ascertaining additional funds to support lasting VBD surveillance and control activities when disease priorities shifted in the community. National and state funding for VBDs did not meet the reported needs for maintaining a robust VBD program. As reported by a participant, *“…There aren’t good funding sources for vector programs. If the county isn’t allocating money, there isn’t state and federal dollars trickling into the local health departments to run these programs*.*”* (Program Coordinator, 100K+) In 2023, 2.3% of the FY 2022–2023 state operating budget was allocated to Public Health and Environment (i.e., $913.5 million dollars) [48]. The Centers for Disease Control and Prevention (CDC) fiscal year (FY) 2023 Grants Summary Profile Report for Colorado describes selected CDC grants and cooperative agreements provided to health departments, universities, and other public and private agencies in Colorado. In 2023, a budget of $7.6 million dollars was allocated to Colorado for Emerging and Zoonotic Infectious Diseases, with VBDs receiving only $1.8 million (24%) of those funds [46]. CDC Public Health Emergency Preparedness reports show funding for fiscal years (FY) 2021–2023 for Colorado remained consistently in the range of $10.2-$10.8 million dollars [45] to support frequent public health emergencies including infectious disease outbreaks. Outside of emergency scenarios, consistent long-term funding was largely reported as being inaccessible and sporadic for VBD programs. Furthermore, interviewees reported that after an emergency concludes federal funding “*goes away*” and the “*infrastructure [is] dissolved because it’s not sustainable*.*”* (Program Manager, 100K+)

#### Importance of engaging in community education and building buy-in.

The importance of building stronger partnerships with health care providers, increasing community engagement, and enhancing public understanding of vector diseases was also highlighted by interviewees. *“… I really, really think a lot of it is that not everyone is aware that* *[VBD] is a danger... [there is a] lack of providers who are literate, especially having tick borne illnesses [in* Colorado*]*.*”* (Epidemiologist, 100K+)

Respondents expressed concern with the lack of public knowledge of VBDs and the dangers surrounding human interaction with wildlife and the potential for increased disease transmission. *“...But I think the community as [a] whole doesn’t understand public health, vector being just one of the many programs dealing with diseases they don’t understand.”* One interviewee mentioned their VBD program had “*no active engagement of the community for them to fully understand some of the [vector-borne] disease and risks.”* (Program Coordinator, 100K+) Survey findings support these assertions with 42% of public health organizations reporting that they do not conduct any community outreach activities.

Respondents also underscored the critical role the community plays in supporting VBD programs and initiatives. *“I think the community is a critical stakeholder and they’re the ones who are trying to act. So again, I would say that the information exchange [from the* public health organization*] with the community is probably the weakest link... I don’t think people always consider the community as a critical stakeholder.”* (Program Coordinator, 100K+) Further, respondents expressed concerns about the community as a potential barrier to funding sources if the public were to withdraw support for VBD containment and mitigation strategies. *“…convincing people that vector-borne illnesses are a priority, especially if they’re worried about other things like economic downturn or childcare. Like, it’s pretty low on most people’s priorities.”* (Infectious Disease Epidemiologist, 100K+)

This was underscored by respondents reporting concern for pesticide use as one main challenge that could lead to the abolishment of VBD surveillance and control programs if discontentment is strong enough. *“So getting one of the biggest, biggest problems that we’re facing now with West Nile is the lack of resources at the local level to conduct evidence-based mosquito control and mosquito surveillance specifically for West Nile vectors. As well as some pushback from communities about using pesticides to spray.”* (Program Manager, 100K+)

#### Cases and negative cases across population sizes.

When comparing cases and negative cases, cases reported a heightened sense of urgency and both internal (e.g., leadership expectations) and external pressure (e.g., community concerns) to address VBDs, whereas negative cases did not report similar drivers to prioritize VBDs. Public health organization cases were mostly from large populations of 100K+ (n = 10; 67%) and expressed concerns about the recent increase in VBDs. The three negative cases represented public health organizations from populations < 100K (<10K, 10-50K, 51-100K). The burden of human cases of VBD in negative case public health organizations were reported in interviews to be low to nonexistent, instead prioritizing surveillance and control activities for foodborne and respiratory illnesses.

Many case interviewees mentioned investigating human cases of reported VBDs with no active vector species surveillance efforts to guide control measures. Cases also mentioned that their communities’ echoed concerns of VBD prioritization especially when observing dead animals in wild (birds) that may carry vector-borne related disease. Negative public health organization cases rarely had reports of human VBD cases in their communities; however, interviewees mentioned they occasionally got reports of human exposure to dead animals, mainly bats, and described an increased incidence of equine WNV in neighboring counties. This did not bring external pressure or concern from their communities for VBDs. Negative case interviewees also highlighted that because they do not perform surveillance of these vectors, they cannot be confident as to whether or not vector species of concern exist in their area.

While both case and negative case interviewees mentioned sending vector species samples to outside testing laboratories, cases mentioned being able to do so with greater support through internal funding and capacity structures. Negative cases reported having limited resources to test samples of animals and vectors for pathogens at an outside laboratory. One negative case interviewee mentioned that their community is in a remote, rural area with geographical barriers to mailing specimens, especially during winter months when snowy conditions can make transportation more difficult. Interviewees from negative case public health organizations mentioned community partners (i.e., veterinarians, the public) may bring in samples for pathogen testing but underscored that the sender is often responsible for the associated cost. To circumvent this, these community public health organizations described applying to grants that will support sample testing kits and fees.

Cases were more likely to report being located in urban settings at lower elevations where humans, infected wildlife, and vectors have the potential to be in close contact. Case interviewees reported human cases of VBDs mostly originating from within their county, with travel acquired cases occurring secondarily. This differs from negative case public health organizations where interviewees reported working in areas with high elevation levels that aren’t currently ecologically suitable for vector species. When human cases were reported within these negative case counties, interviewees noted that they were almost always travel related cases acquired from a different state within the US or from travel outside of the US.

Community education was described as an important measure by both negative case and case interviewees. Negative case interviewees were concerned that strain from the COVID-19 pandemic impacted established relationships with the general public and community institutions (schools, hospitals, nursing homes, jails). They mentioned that the general public may not be aware of the public health organization disease reporting phone lines or that individuals should call in if they have a vector-borne disease exposure. Similarly, cases stressed the importance of educating the public on how to keep themselves and their environment safe from vector-borne diseases and why it is important to be aware of these vectors ability to transmit disease especially during the summer months.

Negative cases mentioned they’ve had “tremendous turnover” of staff in recent years since the COVID-19 pandemic and have found it difficult to attract skilled public health practitioners to their more remote areas. Moreover, multiple negative case interviewees mentioned that they are solely responsible for their infectious disease case investigation and response without “backups” for their roles in either function or disease knowledge. They noted that being the only staff member responsible for these tasks can create a delay in response to investigation if they are out of the office and expressed concern that their public health organizations would lack the capacity to address a future disease outbreak. Case public health organization interviewees reported having a collaborative internal structure that promotes working with other epidemiologists and environmental health specialists to conduct surveillance and control activities.

## Discussion

In Colorado, VBDs have surged in the past two years [5,6,10]. Our mixed methods study of Colorado public health organizations aimed to assess VBD surveillance and control efforts and determine if vector surveillance and control programs are adequately funded and resourced. Our study found that most public health organizations perceived their VBD surveillance and program capacity as inadequate and reported significant limitations in their ability to carry out vector control activities.

Colorado has one of the highest burdens of West Nile fever in the US (2.24 per 100,000 population) [49]. Yet, only 38% of its public health organizations conduct vector surveillance for any species. The low level of insecticide spraying (either in-house or contracted) reported (4 of 24 public health organizations) suggests a gap in VBD control activities and underscores the need for more consistent implementation to better understand factors influencing control practices. Our findings indicated that smaller rural and frontier communities (<100K people) were particularly vulnerable to inadequate VBD organizational capacity and resource availability. Competing priorities for emerging disease highlighted the lack of sustainable funding for vector programs, and resulted in reactionary, rather than proactive, responses to VBDs. As VBDs continue to increase in Colorado, outbreaks of diseases like West Nile fever or plague will likely go undetected without improved surveillance. The findings from our study have implications for where surveillance and control are lacking and highlight gaps, challenges, and opportunities to continue improving and supporting such programs for VBDs (Box 1).

**Box 1. Colorado Public Health Organization Vector Surveillance and Control Program Successes, Gaps, and Challenges**

**Successes**

1. Highly skilled and trained staff with a deep understanding of the complexities and inherent barriers of VBDs
2. Foundational public health infrastructure exists allowing for expansion of vector programs
3. Close partnerships and collaboration between local public health organizations and the state health department
4. Innovative and creative problem solving to address arboviral disease burden across the state, with limited capacity

**Gaps**

1. Analytics needed to understand VBD risk and occurrence
a. Partnerships with academic institutions needed for data sharing and forecasting opportunities
2. Evaluation of different approaches to vector surveillance and control across Colorado local public health agencies
3. Standardization of vector surveillance and control (i.e., minimal reporting standard, threshold for pesticide spraying)
4. Investing in vector-borne human surveillance workforce (e.g., entomology, environmental health, public health) through educational and training opportunities

**Challenges**

1. Robust vector surveillance and pathogen testing are lacking, particularly in rural and frontier communities where staffing is strained
  a. Solution: Wide data sharing of vector surveillance across Colorado local public health agencies through a digital platform
2. Vector control activities and pesticide resistance testing largely absent
  a. Solution: Develop a state-wide threshold for pesticide application or standard protocol for pesticide application contractors
3. Colorado is culturally and ecologically diverse, creating highly localized approaches to combat VBDs making standardized procedures difficult
  a. Solution: Create a working group of Colorado local public health stakeholders to participate in developing acceptable state-wide vector surveillance and control standards
4. Public perception of VBD risk is low and fear of pesticide use related harms is high
  a. Solution: Educational programs needed especially during the vector season
5. Sustainable funding is lacking
  a. Solution: Critical need for strong increases to federal and state funding through legislation. Additional funding streams (i.e., grants or cooperative agreements) should also be sought out

Our findings are also similar to activities in other states. A study of 73 Florida mosquito control program capabilities found only 25.0% of respondents sent mosquito pools for testing and noted most arboviral surveillance (serology and pool testing) was conducted at a state laboratory [50]. Monitoring disease risk through mosquito surveillance and pathogen testing is a vital public health function. Other studies have found proactive arboviral surveillance of mosquitos helped prevent outbreaks of WNV disease [51]. Although continuous year-round surveillance is a long-term goal of many public health organizations, periodic monitoring of vectors in the interim can still improve early pathogen detection and inform local risk assessments.

A priority gap in Colorado vector programs is the need for analytics, and digital data sharing of vector and human case surveillance to inform VBD risk and occurrence across Colorado communities. In the absence of vector surveillance data, public health organizations may consider partnering with academic institutions and/or member organizations (e.g., Council for State and Territorial Epidemiologists, National Association of County and City Health Officials) to assist in data acquisition, and collaborative analyses. Furthermore, development of a state or regional forecasting group would provide the opportunity for risk mapping, forecasting case incidence and cross-species transmission as well as informing real-time decision making related to planning and resource allocation [52]. Public health-academic partnerships could also provide smaller or rural public health organizations with additional analytical insights without adding to their burden of work. An ultimate goal could be to have early warning systems in place to model impending risk for VBD prior to detection of VBD activity by surveillance programs [53] and develop fine spatial scale (city blocks or neighborhoods), short-term planning models (days–weeks) for control efforts [54].

We found substantial differences in surveillance and control activities and resources between urban and rural/frontier public health organizations. Agricultural practices in rural regions can drive pesticide resistance [55] and complicate disease prevention, especially in the absence of adequate vector monitoring and control. This presents an opportunity for standardizing vector surveillance and control through minimal reporting standards and pesticide thresholds [56] for spraying which would ensure uniformity across the state and limit pesticide resistance. It is important to first evaluate different approaches currently underway across public health organizations and form a working group of Colorado local public health stakeholders to participate in developing acceptable state-wide vector surveillance and control standards. Resources for building local mosquito control programs through surveillance and control are available through NAACHO [57].

A consistent challenge identified from our study was the low public perception of risk of VBDs as well as the complex task of combating public health skepticism and misinformation. Prior research and modeling studies have shown that simple education campaigns are a low-cost, high impact tool, and may be particularly useful for public health organizations that have limited vector-spraying capacity [58]. The most effective public health messaging approaches ensure messaging is focused on both vector-borne disease transmission awareness as well as general bite prevention through personal protection [59]. Even in settings with high community level vector control, concern of personal risk for being bitten significantly reduced the peak of outbreaks, and the total proportion infected [59]. Local heatmaps offer another opportunity for public health organizations to communicate risks with the general public as well as placing messaging where individuals are at a high risk of VBD exposure, such as trailheads, lakes, and parks [60]. Repeated messaging strategies accumulate to impact individuals’ knowledge and risk perception and behaviors to prevent VBDs [60].

Many public health organizations with consistent funding were from urban areas and conducted the most vector-borne disease activities, whereas the rural/frontier public health organizations had limited support and were limited in their VBD activities. Differences across urban vs rural surveillance capacity are well known but also highlight the need to support public health organizations servicing rural/frontier populations. A Southeastern US study found public health agencies servicing larger communities were three times more likely to perform pathogen testing and four times more likely to perform year-round surveillance compared to agencies servicing below 100K residents [61]. Like many rural communities where healthcare access can be challenging, Colorado’s landmass is predominantly rural or frontier [62], and delays in outbreak response to VBDs in rural regions can also result in delayed detection and treatment of cases, resulting in more severe disease outcomes. Without strengthened investment in rural/frontier communities, persistent resource inequities may leave these communities more vulnerable to emerging VBD threats.

Public health organizations often encountered threats to funding and reprioritization of other diseases, which contributed to a critical lack of staff required to implement or expand vector-borne disease surveillance and control. Public health organizations reported difficulty in attracting staff with experience conducting disease surveillance. Investing in VBD vector and human surveillance workforce (e.g., entomology, environmental health, public health) through educational and training opportunities is a key gap found through our research. Regional training and evaluation centers that create VBD specific curriculum, trainings, and summer internships should be integrated into state and local public health as they provide an opportunity for interested students to learn from established professionals in the field and create a pipeline of future workers [63,64].

Lasting funding structures at the federal and state level are critical to supporting ongoing VBD programs. While the FY2024–25 Colorado Joint Budget Committee Hearing was noted to have enabled the Colorado Department of Public Health and Environment to hire a medical entomologist to track insect disease vectors, monitor for emerging VBDs in Colorado, and conduct trainings and consult with local partners [65] state budgets have not publicly itemized specific funding for VBD [66] since FY 2011–2012, when the Vector Program was last explicitly listed. [67]. Despite possible improvements to VBD state programs through federal funding distributions (e.g., CDC) [68], our findings indicate that Colorado public health organizations still lack critical resources and capacity for VBD surveillance and control.

Despite the challenges, many public health organizations showed a number of successes in the face of adversity. Many of the representatives interviewed had a deep understanding of the local complexities of VBDs in their area, and innovative and creative solutions like the use of drone vector surveillance, standardizing control strategies across their county, and engaging local high school students in vector surveillance through summer internship programs. If funding for VBDs were to improve, public health practitioners and infrastructure of many organizations are ready for expansion. From our interviews, it was clear that many participating organizations had close partnerships and collaborations with other interdisciplinary local agencies, bordering county public health organizations, as well as with the state health department. Many interviewees were enthusiastic and optimistic about the implementation of EpiTrax, designed to integrate multiple data sources and deliver real-time disease trend data to support public health surveillance and response efforts.

Strengths of this study included a mixed methods case study design that enhanced the scope and complexity of our research by bringing the strengths of both qualitative and quantitative methods to identify contextual factors at public health organizations associated with VBD surveillance and control. A key strength of our assessment was our ability to include public health organizations from high burden WNV areas where surveillance needs are greatest. Additionally, this study represents one of the few mosquito and control evaluations focused on the Rocky Mountain region with insights into the Western US outside of California – a commonly studied state for arboviral disease. Our study has some limitations. First, our study was likely impacted by selection bias as respondents who opted to participate may have been more engaged than those who did not participate. Similarly, non-response bias from those failing to identify themselves in the survey was a concern, but there did not appear to be differences when comparing the characteristics of those who failed to include their name in the survey to those who did include their name. Second, survey and interview responses were not regionally representative of all Colorado public health organizations and disproportionally reflected urban public health organizations. Therefore, our results may be biased toward the experiences and challenges of public health organizations in urban settings with less representation of public health organizations in rural and frontier regions, limiting the generalizability of our results.

Future studies should conduct a probability-based sampling strategy [34] and invest in pre study engagement with rural public health leaders and associations to establish relationships and recognition. Furthermore, it will be important to consider the burden of a lengthy interview as a participation barrier and whether curtailing the interview timing may enhance rural involvement. Finally, our study framework did not evaluate public health capacity related to pathogen and vector ecology surveillance, or considerations related to reservoir hosts, but future studies on individual VBDs should also consider ecological aspects as part of their evaluation.

Future directions include expanding research evaluating the disparities between rural and urban communities in vector-borne programs to understand the implications of increased funding and resource support on VBD outcomes in these communities. Additional studies should also evaluate the impacts of climate change on VBDs and its impacts on VBD surveillance, control, and response efforts in Colorado. Shifts in climate can heighten arbovirus vector adaptability and underscore the importance of routine surveillance for mosquito vectors. Future research will aim to strengthen our understanding of the dynamics and seasonality of vector species in this region and public health organization preparation efforts and climate mitigation strategies.

In conclusion, our mixed methods study highlights the need for enhanced analytic capacity to better characterize vector risk and disease occurrence, strengthened partnerships between public health organizations and academic institutions, standardizing minimum reporting requirements and evidence-based thresholds for control activities, and a sustained investment in the VBD related workforce. The expansion of VBDs into higher elevations and rural areas will require significant investment in funding mechanisms for smaller communities. Continued assessments of vector surveillance and control activities by organizations and researchers will be critical to guide policy makers as to why sustained funding is needed, particularly in a funding environment that is growing more challenging from year to year. Strengthening these core capacities is critical to protecting communities across Colorado, especially those most vulnerable to environmental and VBD health threats.

## Supporting information

S1 Text Supporting Methodology and Results. (DOCX)

S1 Appendix Mosquito and Vector Control Agency Capacity and Needs Assessment Survey for Emerging Vector-Borne Disease. (DOCX)

S2 Appendix 2024 Colorado Health Department Vector Borne Program Needs Assessment. Interview Summary Sheet and Guide.
(DOCX)

S1 Data Deidentified Dataset of Survey Responses. (XLSX)

S1 Fig 2023 West Nile Virus Cases and Participating Public Health Organization Regions. (TIF)

S2 Fig Surveillance and Control MCA Scree Plot and Variable Map. (TIF)

S3 Fig Resource Barriers MCA Scree Plot and Variable Map. (TIF)
