# Correction: Vector-borne disease surveillance and control resource needs in Colorado public health organizations

**DOI:** 10.1371/journal.pone.0353937

**Published:** 2026-07-14

**Authors:** Jessica L. Butler, Jordan A. Crawford, Kayleigh P. Keller, Thomas Jaenisch, Molly M. Lamb, Juliana G. Barnard, Talia M. Quandelacy

The S1 Text was uploaded incorrectly. Please view the correct S1 Text below.

## Supporting information

S1 Text Supporting Methodology and Results. (DOCX)
