# Supplementary material for: Vector-borne disease surveillance and control resource needs in Colorado public health organizations
Source: PLoS One. 2026 Apr 20;21(4):e0347142. doi: 10.1371/journal.pone.0347142 (PMC13095035; doi:10.1371/journal.pone.0347142)
Supplement: S1 Appendix — (DOCX) [file pone.0347142.s002.docx]

Mosquito and Vector Control Agency Capacity and Needs Assessment for Emerging Vector-Borne Disease

You are being asked to participate in this research study because you were identified as a public health leader, data analyst, epidemiologist, or administrator who has firsthand knowledge of vector borne disease programs. We are interested in individuals who have job functions including direct vector borne disease surveillance responsibilities such as collection, management, analysis, or response.

If you join the study, you will complete a survey consisting of 45 multiple choice questions and free text-response questions. The survey will take approximately 10-20 minutes to complete via Qualtrics.

This study is designed to learn more about vector borne resource availability.

Possible discomforts or risks include loss of time. There may be risks the researchers have not thought of.

This study is not designed to benefit you directly.

Every effort will be made to protect your privacy and confidentiality by deidentifying and aggregating the data so that no one individual is identifiable.

You have a choice about being in this study. You do not have to be in this study if you do not want to be.

The data we collect will be used for this study but may also be important for future research. Your data may be used for future research or distributed to other researchers for future study without additional consent if information that identifies you is removed from the data.

If you have questions, you can call Jessica Butler at (786) 277-2934. You can call to ask questions at any time.

You may have questions about your rights as someone in this study. If you have questions, you can call COMIRB (the responsible Institutional Review Board) at (303) 724-1055.

By completing this survey, you are agreeing to participate in this research study.

Do you consent to participate in this research study?

- Yes (1)
- No (2)

Skip To: End of Survey If Do you consent to participate in this research study? = No

End of Block: Block 1

Start of Block: Default Question Block

Thank you for your time and interest! This questionnaire is confidential, and your participation is voluntary. The questionnaire should take less than 20 minutes to complete.

Your answers will help us inform policymakers as to the needs of local vector control agencies in order to strengthen the overall vector borne disease management and capacities within Colorado. Responses will be deidentified and your name will not be linked to specific responses or comments. Your responses will be summarized and aggregated with others.

| Page Break |  |
| --- | --- |

Q1 What is your name?

________________________________________________________________

Q2 For which type of public health agency do you work? (Select one)

- City (1)
- County (2)
- State (3)
- Other (4) __________________________________________________

Q3 In what setting is your organization located? (Select one)

- Urban (population of at least 10,000 inhabitants) (1)
- Rural (population less than 10,000 inhabitants) (2)
- Frontier (population density of six or fewer persons per square mile) (3)
- Not applicable (4)

Q4 What is the estimated population size of the residents you serve? (Select one)

- (1)
- 10-50,000 (2)
- 51-100,000 (3)
- 100,000+ (4)
- Don’t know/unsure (5)

Q5 Please choose the title that best represents your job role. (Select one)

- Epidemiologist (1)
- Data Analyst (2)
- Laboratory Technician (3)
- Communication Specialist (4)
- Policy Analyst (5)
- Administrator (6)
- Other (7) __________________________________________________

Q6 Please choose the job responsibilities that best represent your job. (Select all that apply)

- Disease surveillance (1)
- Disease control (2)
- Data management (3)
- Laboratory analysis (4)
- Community outreach (5)
- Public education (6)
- Policy (7)
- None of these (8)

Q7 What is the highest level of education you have completed? (Select one)

- High school diploma or equivalent (1)
- Bachelor’s degree (2)
- Master’s degree (3)
- Doctoral or professional degree (MD, DO, PharmD, etc.) (4)

Q8 How many years of work experience in vector borne disease do you have? (Select one)

- 0-5 years (1)
- 6-10 years (2)
- 10+ years (3)
- Not applicable (4)

| Page Break |  |
| --- | --- |

Q9 **The next series of questions are related to vector borne surveillance activities within your current organization.**

Q10 Which vector borne diseases have been reported in your county in the previous 12 months? (Select all that apply)

- West Nile virus (1)
- Eastern equine encephalitis (2)
- LaCrosse encephalitis (3)
- St. Louis encephalitis (4)
- Spotted fever group rickettsiosis (5)
- Lyme disease (6)
- Murine typhus (7)
- Chagas disease (8)
- Dengue virus (9)
- Plague (10)
- Tularemia (11)
- None (12)
- Other (13) __________________________________________________
- Don’t know/unsure (14)

Q11 Does your organization conduct their own vector surveillance? (Select one)

- Yes (1)
- No (2)
- Don't know/unsure (3)

Skip To: Q13 If Does your organization conduct their own vector surveillance? (Select one) = No

Q12 What type of surveillance does your organization conduct during the year? (Select one)

- Vector collections (1)
- Pathogen testing (2)
- Other (3) __________________________________________________
- Don't know/unsure (4)
- Not applicable (5)

Q13 When does your organization conduct surveillance during the year? (Select one)

- Summer (1)
- All year (2)
- Other (3) __________________________________________________
- Don't know/unsure (4)
- Not applicable (5)

Q14 Is there someone in your organization that performs geographic information systems (GIS) or mapping services?  (Select one)

- Yes (1)
- No (2)
- Don't know/unsure (3)

| Page Break |  |
| --- | --- |

Q15 **The next series of questions are related to vector borne control activities within your organization.**

Q16 For which vector(s) has your organization conducted control activities for in the previous 12 months? (Select all that apply)

- Mosquitos (1)
- Ticks (2)
- Kissing bugs (3)
- Bed bugs (4)
- Sandflies (5)
- Fleas (6)
- Pests (spiders, rats, etc.) (7)
- None (8)
- Other (9) __________________________________________________
- Don’t know/unsure (10)

Q17 Does your organization perform insecticide spraying for any vector? (Select one)

- Yes (1)
- No (2)
- Don't know/unsure (3)

Skip To: Q28 If Does your organization perform insecticide spraying for any vector? (Select one) = No

Q18 With what frequency does your organization apply insecticides for each vector during high season? (Select one response per row)

|  |  |
| --- | --- |
| Mosquitos (1) | ▼ Daily (1) ... Not Applicable (6) |
| Ticks (2) | ▼ Daily (1) ... Not Applicable (6) |
| Kissing Bugs (3) | ▼ Daily (1) ... Not Applicable (6) |
| Bed Bugs (4) | ▼ Daily (1) ... Not Applicable (6) |
| Sandflies (5) | ▼ Daily (1) ... Not Applicable (6) |
| Fleas (6) | ▼ Daily (1) ... Not Applicable (6) |
| Pests (spiders, rats, etc.) (7) | ▼ Daily (1) ... Not Applicable (6) |

Display This Question:

If With what frequency does your organization apply insecticides for each vector during high season?... = Other

Q18 If other, please elaborate.

________________________________________________________________

Q19 How is insecticide application conducted within your organization? (Select all that apply)

- In-house (1)
- Contractor (2)
- Other (3) __________________________________________________
- Don’t know/unsure (4)

Q20 How does your organization apply insecticides? (Select all that apply)

- Organization-owned truck (1)
- Organization-owned aerial (2)
- Hand spraying (3)
- Other (4) __________________________________________________
- Don’t know/unsure (5)

Skip To: Q22 If How does your organization apply insecticides? (Select all that apply) = Don’t know/unsure

Q20 How many trucks does your organization own that are equipped for spraying insecticides? (Select one)

- 0-5 (1)
- 6-10 (2)
- 10+ (3)
- Not applicable (4)
- Don’t know/unsure (5)

Q21 How many airplanes and/or helicopters equipped for spraying insecticides does your organization own? (Select one)

- 0-2 (1)
- 3-5 (2)
- 6+ (3)
- Not applicable (4)
- Don’t know/unsure (5)

Q22 Does your organization follow a vector threshold response level (sufficient density of competent vectors above the threshold) for implementing insecticide spraying within your catchment area? (Select one)

- Yes (1)
- No (2)
- Don’t know/unsure (3)

Q23 What treatment thresholds does your organization abide by? (Select all that apply)

- Presence of nuisance insects (such as fleas, ticks, spiders, chiggers, earwigs, ants, slugs, snails, tent caterpillars, and/or centipedes) (1)
- Positive mosquito pool(s) (2)
- Positive human case(s) (3)
- Positive avian case(s) (4)
- Positive equine case(s) (5)
- Pre-determined schedule (6)
- Other (7) __________________________________________________
- Don’t know/unsure (8)

Q24 Does your organization use adulticides? (Select one)

- Yes (1)
- No (2)
- Don’t know/unsure (3)

Skip To: Q26 If Does your organization use adulticides? (Select one) = No

Q25 Which adulticides does your organization use? (Select all that apply)

- Coils (1)
- Foggers (2)
- Yard sprays or aerosols (3)
- Other (4) __________________________________________________
- Don’t know/unsure (5)

Q26 Does your organization use larvicides? (Select one)

- Yes (1)
- No (2)
- Don’t know/unsure (3)

Skip To: Q28 If Does your organization use larvicides? (Select one) = No

Q27 What larvicides does your organization use? (Select all that apply)

- Biological control (1)
- Growth regulators (2)
- Contact insecticides (3)
- Stomach insecticides (4)
- Other (5) __________________________________________________
- Don’t know/unsure (6)

Q28 Are mosquitoes with Wolbachia available within your catchment area as a mosquito control measure? (Select one)

- Yes (1)
- No (2)
- Don't know/unsure (3)

| Page Break |  |
| --- | --- |

Q29 **The next series of questions are related to vector borne laboratory testing and evaluation practices within your organization.**

Q30 How does your organization perform infectious disease testing? (Select all that apply)

- In-house (1)
- Send to an outside laboratory (outside state and/or in-state contracting lab) (2)
- Testing not performed (3)
- Other (4) __________________________________________________
- Don’t know/unsure (5)

Q31 Is there a lab technician or microbiologist in your organization that performs insecticide resistance testing? (Select one)

- Yes (1)
- No (2)
- Don't know/unsure (3)

Skip To: Q35 If Is there a lab technician or microbiologist in your organization that performs insecticide resist... = No

Skip To: Q32 If Is there a lab technician or microbiologist in your organization that performs insecticide resist... = Yes

Q32 How is the resistance testing conducted? (Select one)

- Field cage tests (1)
- Bottle bioassays (2)
- Other (3) __________________________________________________
- Don’t know/unsure (4)

Q33 How frequently does your organization test for insecticide resistance? (Select one)

- Once a year (1)
- Twice per year (2)
- Three or more times per year (3)
- Don’t know/unsure (4)

| Page Break |  |
| --- | --- |

Q34 **The next series of questions are related to preparedness and response efforts to** climate-related vector borne disease threats **within your organization.**

Below we use the terms climate risk and climate vulnerability.
 **Climate risk** is the potential for negative consequences for human or ecological systems from the impacts of a changing climate.
 **Climate vulnerability** describes the degree to which natural, built, and human systems are at risk of exposure to impacts from a changing climate.

Q35 In your opinion, is your agency **prepared** for a natural disaster-related vector borne disease threat (e.g., heavy summer rains driving mosquito populations)? (Select one)

- Yes (please elaborate) (1) __________________________________________________
- No (2)
- Don’t know/unsure (3)

Q36 In your opinion, is your agency equipped with the **knowledge** to manage disaster risk reduction and adaption to a changing climate at a community or neighborhood level? (Select one)

- Yes (please elaborate) (1) __________________________________________________
- No (2)
- Don’t know/unsure (3)

Q37 In your opinion, is your agency equipped with the **experience** to manage disaster risk reduction and adaption to a changing climate at a community or neighborhood level? (Select one)

- Yes (please elaborate) (1) __________________________________________________
- No (2)
- Don’t know/unsure (3)

Q38 In your opinion, is your agency equipped with the **resources** to manage disaster risk reduction and adaption to a changing climate at a community or neighborhood level? (Select one)

- Yes (please elaborate) (1) __________________________________________________
- No (2)
- Don't know/unsure (3)

Q39 Are there existing partnerships between your organization, the community, and/or healthcare organizations to reduce climate-related risks (e.g., storms, flooding, wildfires)? (Select one)

- Yes (please elaborate) (1) __________________________________________________
- No (2)
- Don't know/unsure (3)

Q40 Are there existing partnerships between your organization, the community, and/or healthcare organizations to reduce climate-related vulnerability (e.g., water scarcity, incidents of infectious diseases) in surrounding communities? (Select one)

- Yes (please elaborate) (1) __________________________________________________
- No (2)
- Don't know/unsure (3)

Q41 Is there someone in your agency that performs climate-related outreach and education to the community you serve? (Select one)

- Yes (1)
- No (2)
- Don't know/unsure (3)

Skip To: Q42 If Is there someone in your agency that performs climate-related outreach and education to the commu... = Yes

Skip To: Q43 If Is there someone in your agency that performs climate-related outreach and education to the commu... = No

Q42 Based on the previous question, what type of outreach/education is performed? (Select all that apply)

- School-based programs (1)
- Media (magazine, news outlets) (2)
- Social media (3)
- Website/online presence (4)
- Mobile units (5)
- Other (6) __________________________________________________
- Don’t know/unsure (7)

Q43 Does your organization participate in community educational programs to assist the local community in reducing climate risk and vulnerabilities? (Select one)

- Yes (please elaborate) (1) __________________________________________________
- No (2)
- Don't know/unsure (3)

Q44 In the event of a natural disaster (e.g., wildfire, flooding, tornados), how timely are funds and/or resources available from the state of Colorado to your local public health agency? (Select one)

- Within 24 hours (1)
- Within 72 hours (2)
- Not at all (3)
- Other (4) __________________________________________________
- Don’t know/unsure (5)

| Page Break |  |
| --- | --- |

Q45 **The next series of questions are related to vector borne resource availability within your organization.**

Q46 What are additional **testing methods** that you would like to use in your organization, but do not have the resources for? (Select all that apply)

- Insecticide resistance testing (1)
- Pathogen testing (2)
- Other (3) __________________________________________________
- Don’t know/unsure (4)

Q47 What are additional **control methods** that you would like to use in your organization, but do not have the resources for? (Select all that apply)

- Additional chemical methods (1)
- Additional biological methods (2)
- Other (3) __________________________________________________
- Don’t know/unsure (4)

Q48 Is your **testing** equipment up-to-date and reliable? (Select one)

- Yes (1)
- No (2)
- Not applicable (3)
- Don't know/unsure (4)

Q49 Is your insecticide **application** equipment up-to-date and reliable? (Select one)

- Yes (1)
- No (2)
- Not applicable (3)
- Don't know/unsure (4)

Q50 What is the **most important barrier**you see for vector borne disease prevention and control in your organization? (Select one)

- Resources (1)
- Personnel Funds (2)
- Training (3)
- Other (4) __________________________________________________
- Don't know/unsure (5)

Q51 What is the **most important type** of training needed in your organization? (Select one)

- Vector surveillance (1)
- Vector testing (2)
- Insecticide resistance evaluations (3)
- Vector control (4)
- Other (5) __________________________________________________
- Don’t know/unsure (6)

Q52 What is the **most optimal method** of training dissemination for your organization? (Select one)

- In person, onsite training (1)
- Online webinar (2)
- Multiple day workshop, offsite (3)
- Other (4) __________________________________________________
- Don't know/unsure (5)

Q53 Thank you for your participation! Are there any final thoughts you would like to share with us?

________________________________________________________________

End of Block: Default Question Block
