# Supplementary material for: Vector-borne disease surveillance and control resource needs in Colorado public health organizations
Source: PLoS One. 2026 Apr 20;21(4):e0347142. doi: 10.1371/journal.pone.0347142 (PMC13095035; doi:10.1371/journal.pone.0347142)
Supplement: S2 Appendix — Interview Summary Sheet and Guide. (DOCX) [file pone.0347142.s003.docx]

**2024 Colorado Health Department Vector Borne Program Needs Assessment**

**Interview Summary Sheet and Guide**

Interviewer Name: ______

Recorder Name: ____________________________

Date: ________________

Interviewee Name: ______________________________

Job Title: ______________________________________

Colorado County: ______________

Data Entry Date: _____________

Data Entry By: ________________

**Main Research Question:**

**Explore resource needs, organizational capacity, and implementation barriers to climate specific tools and mitigation strategies to vector borne diseases within Colorado state and local departments of public health.**

Hello, my name is __________________, it’s nice to meet you.

Thank you for taking the time to meet with me and agreeing to participate in this Colorado Vector-Borne Disease Program Needs Assessment.

We are interested in receiving your honest responses to these questions to better understand the resource needs within Colorado’s departments of health and implementation barriers to vector-borne disease surveillance and control.

Please tell me if any questions need clarification or additional explanation during the interview. We anticipate the interview will take approximately 45 minutes.

We have a set of questions we will be asking. I will be recording your responses and comments, so that the information can be combined with the responses of the other interview participants.

Your responses will be summarized and aggregated with others. Responses will be deidentified and your name will not be linked to any specific responses or comments.

You are free to stop the interview at any time.

Are you ready to begin?

I will start recording the session.

1. Can you tell me about your role at your < health department> and current day-to-day activities?

For this interview, vector-borne diseases will refer to diseases caused by both mosquito and arthropod-borne pathogens (inclusive of ticks and fleas).

1. Are vector borne diseases (VBD) a priority in your <health department>?
2. What would you say are the top three issues specific to VBDs that your <health department> is most concerned about?
3. What do you think are the strongest assets of how your <health department> addresses VBDs in your community?

The next series of questions will ask about resource availability within your < health department>.

1. Are there technological tools or innovations that would be useful to have access to that you currently do not have in your role?
   1. If so, what barriers exist that prevent you from having access to these tools?
2. Can you tell me about the most recent training you took for career development?

The next series of questions will ask about the organizational structure and capacity within your <health department>.

1. What practices or processes are in place to ensure <health department> can adequately respond to health needs of the community you serve?
2. What do you think are resource and capacity challenges to addressing VBDs in your community?
3. If you had a magic wand, what top initiatives would you implement within your VBD surveillance and control activities <health department> over the next three years?
4. What state or local partnerships does your <health department> have to **prevent** VBDs?
5. Probes for regional mosquito control associations, academic institutions, other government agencies.
6. What state or local partnerships does your <health department> have to **control** VBDs?
7. Who do you see as key stakeholders in the implementation of VBD program activities?

The final series of questions will ask about how your <health department> plans to prepare for a changing climate in Colorado.

1. What does your <health department> do to address the impact of the changing climate on VBD prevention and control within your <county/state>?
2. Does your <health department> have the capacity to identify areas in the community that may be at risk for climate impacts related to VBD (ex. heavy summer rains driving mosquito populations, and subsequent tick-borne disease outbreaks)?

That concludes our structured series of questions.

1. Was there anything else related to VBDs activities you wanted to discuss today that we did not ask?
2. Do you have any questions for us?
3. Are you interested in receiving the results of this study?
4. Are there other colleagues that work at your <health department> that might be interested in participating in this study?

Thank you for your time. We appreciate your participation and willingness to share insights.
