# Supplementary figures and images for: Vector-borne disease surveillance and control resource needs in Colorado public health organizations
Source: PLoS One. 2026 Apr 20;21(4):e0347142. doi: 10.1371/journal.pone.0347142 (PMC13095035; doi:10.1371/journal.pone.0347142)

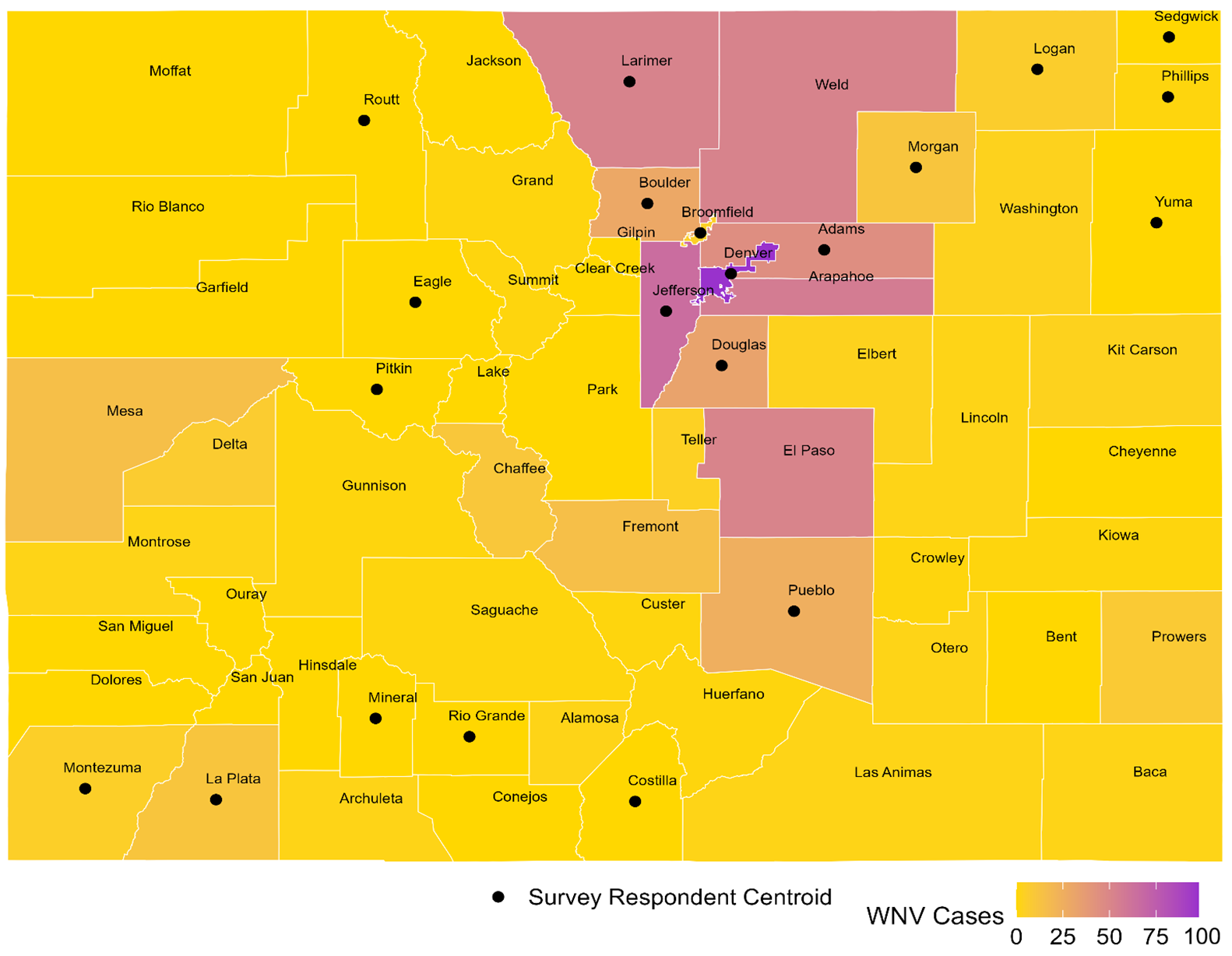

Supplement: S1 Fig — (TIF) [file pone.0347142.s005.tif]

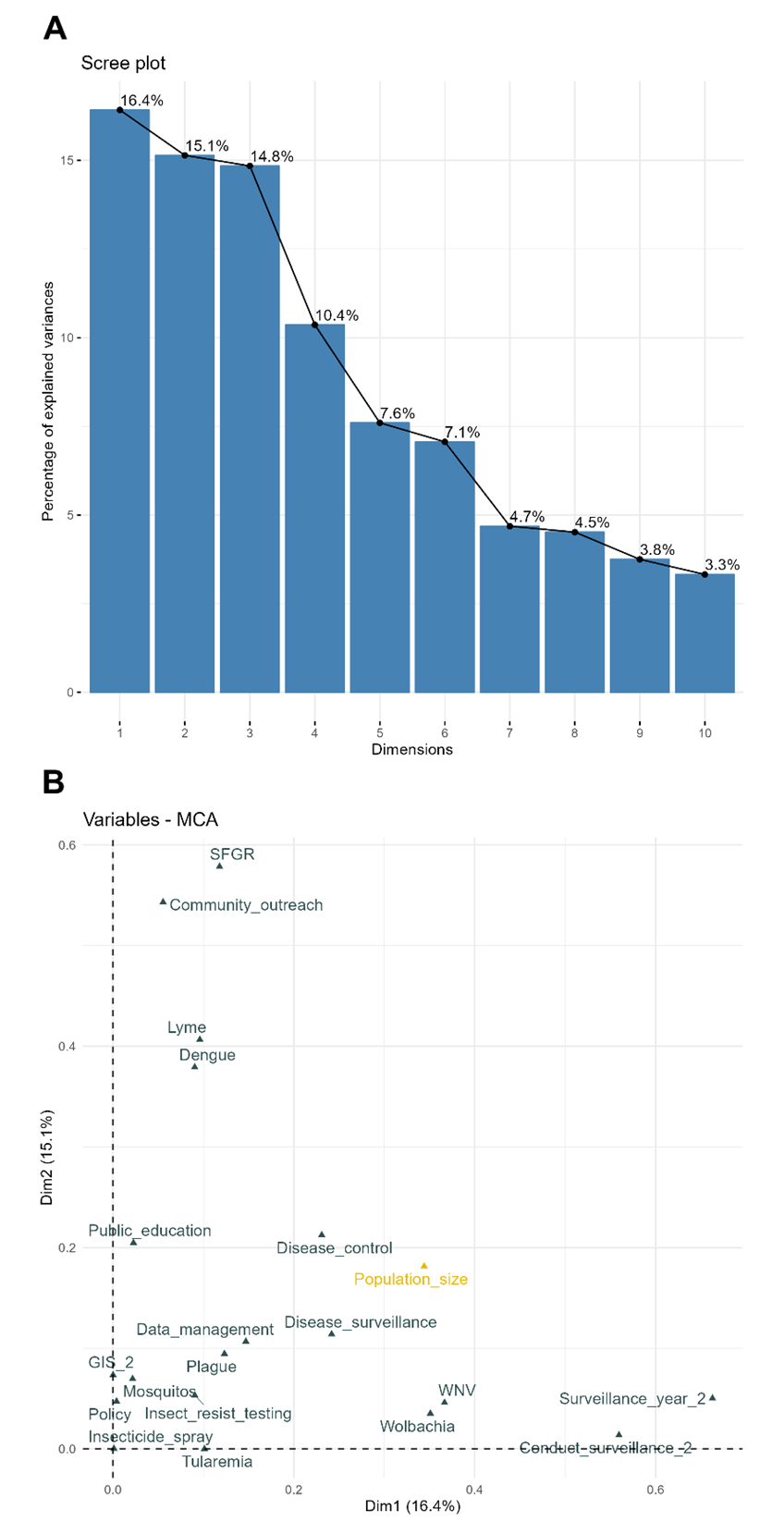

Supplement: S2 Fig — (TIF) [file pone.0347142.s006.tif]

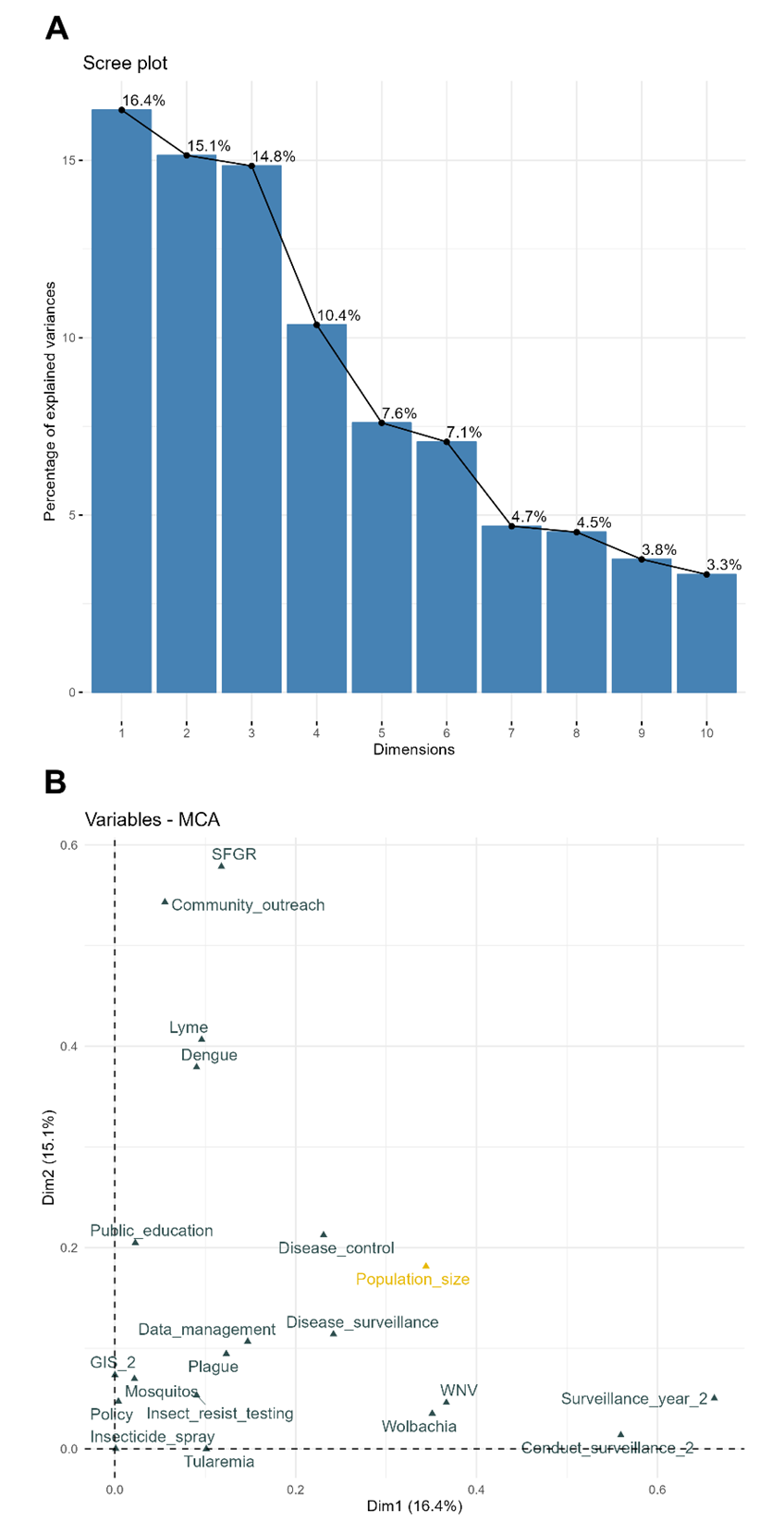

Supplement: S3 Fig — (TIF) [file pone.0347142.s007.tif]
