# Supplementary material for: Correction: Vector-borne disease surveillance and control resource needs in Colorado public health organizations
Source: PLoS One. 2026 Jul 14;21(7):e0353937. doi: 10.1371/journal.pone.0353937 (PMC13367695; doi:10.1371/journal.pone.0353937)
Supplement: S1 Text — (DOCX) [file pone.0353937.s001.docx]

**Supporting Information**

**Methods**

We collected annual county-level WNV cases in Colorado from the Colorado Department of Public Health and Environment [1]. We created spatial maps to show the 2023 WNV cases and survey respondents across Colorado counties using US Census TIGER/Line Shapefiles for Colorado counties [2].

To examine relationships among the survey responses, we conducted a multiple correspondence analysis (MCA) on two overarching themes: surveillance and control activities; and VBD resources. We categorized multi-level response variables into binary variables in preparation for the MCA. Variables were meaningfully collapsed when responses had <5 individuals per level. Surveillance and control survey variables were included as active, meaning their categories were used to define the dimensions or axes of the MCA. Sub-analyses evaluated population size (<10,000; 10,000-50,000; 51,000-100,000; 100,000>), location, and county type as additional components. A supplementary variable (population) was included to examine the relationship with active variables but not to influence the primary structure of the dimensions created by the active variables. We calculated the Spearman correlation coefficient to assess the collinearity of these three variables and population size was selected to represent the population-stratified indicator in sub-analyses

**Results**

In 2023, the annual WNV incidence rates ranged from 0.07 to 0.38 per 1,000. Morgan County saw the highest rates of WNV (0.38 per 1,000) in 2023 of counties included in our study (S1 Fig). Costilla, Eagle, Pitkin, Rio Grande, Sedgwick and Yuma Counties had no reported WNV cases.

**Supplemental Figure 1. 2023 West Nile Virus Cases and Participating Public Health Organization Regions.** Colors show WNV cases across Colorado counties. Points reflect the county centroid of the participating public health organizations.

When evaluating clustering of factors related to vector surveillance and control from survey responses (S1 Table), the MCA identified six dimensions that explained 71.4% of the variance (S2 Fig).

**Supplemental Table 1. Surveillance and Control Multiple Correspondence Analysis Question, Label and Responses.**

| **Survey Question** | **Variable As Listed in MCA** | **Response** |
| --- | --- | --- |
| Plague within past 12 months | Plague | Yes; no |
| SFGR within past 12 months | SFGR | Yes; no |
| Lyme within past 12 months | Lyme | Yes; no |
| WNV within past 12 months | WNV | Yes; no |
| Dengue within past 12 months | Dengue | Yes; no |
| Tularemia within past 12 months | Tularemia | Yes; no |
| Mosquitos within past 12 months | Mosquitos | Yes; no |
| Public education activities | Public_education | Yes; no |
| Disease control activities | Disease_control | Yes; no |
| Data management activities | Data_management | Yes; no |
| Disease surveillance activities | Disease_surveillance | Yes; no |
| Policy activities | Policy | Yes; no |
| Community outreach activities | Community_outreach | Yes; no |
| Is GIS conducted at the LHD | GIS_2 | Yes; no |
| Is insecticide resistance testing done | Insect_resist_testing | No; don't know |
| Is insecticide spray used | Insecticide_spray | Yes; no |
| Is Wolbachia technology used | Wolbachia | No; don't know |
| Does LHD conduct surveillance | Conduct_surveillance_2 | Yes; no |
| At what point of the year is surveillance conducted | Surveillance_year_2 | All year; summer |
| Population Size of public health organization area serviced | Population_size | <10K, 10-50K, 51-100K, 100K+ |

**Supplemental Figure 2. Surveillance and Control MCA Scree Plot and Variable Map.** *Panel A shows the proportion of variance explained by the first ten dimensions. Panel B shows the variable map for dimensions 1 and 2. Active variables in dark grey, supplemental variable in yellow. These two dimensions account for 31.5% of the total variance. Distance from the axis indicates the association of the variable to the dimension, where two points that are close to each other have greater association with each other.*

The highest percentage of the variance was explained by dimensions 1 (16.4%) and 2 (15.1%) (S2 Fig). Based on the variable map (S2 Fig) that visualizes the correlation between variables and MCA principal dimensions (S2 Fig), dimension 1 separated respondents primarily based on established surveillance activities. Surveillance activities were associated with whether a public health organization conducts surveillance, conducts surveillance all year round, and had WNV absence within the previous 12 months. Dimension 2 primarily separated respondents based on identification of less established VBDs and community outreach activities. The variables SFGR, Lyme disease, DENV, community outreach and public education are the most correlated with dimension 2.

When evaluating clustering of factors related to resources from survey responses (S2 Table), the MCA identified four dimensions that explained 63.9% of the variance (S3 Fig).

**Supplemental Table 2. Resources Multiple Correspondence Analysis Question, Label and Responses.**

| **Question** | **Variable As Listed in MCA** | **Response** |
| --- | --- | --- |
| Barriers to VBDs | Barrier_VBD_2 | Resources, Personnel Funds, Other |
| Training types | Training_type_2 | Vector surveillance, vector testing, vector control, insecticide resistance evaluations, other, don’t know/unsure |
| Testing Equipment | Testing_equip_2 | No, Yes, Not applicable |
| Pathogen Testing | Pathogen_testing | Yes, No |
| Is insecticide resistance testing done | Insecticide_Resistant_Test | Yes, No |
| Outside Lab available to send samples | Outside_Lab | Yes, No |
| Insecticide application equipment up to date and reliable | Insect_app_2 | Yes, Not Applicable |
| Population Size of public health organization area serviced | Population_size | <10K, 10-50K, 51-100K, 100K+ |

**Supplemental Figure 3. Resource Barriers MCA Scree Plot and Variable Map.** Panel A shows the proportion of variance explained by the first ten dimensions. Panel B shows the variable map for dimensions 1 and 2. Active variables in dark grey, supplemental variable in yellow. These two dimensions account for 43.1% of the total variance. Distance from the axis indicates the association of the variable to the dimension, where two points that are close to each other have greater association with each other.

The highest percentage of the variance was explained by dimensions 1 (24.1%) and 2 (19%) (S3 Fig). Dimension 1 primarily separated respondents based on having reliable insecticide application and a need for insecticide resistance testing (S3 Fig). Requests for pathogen testing equipment and noting important resource barriers and training needs had the biggest impact on illustrating resources at public health organizations for dimension 2 (S3 Fig).

**References**

1. West Nile virus data | Department of Public Health & Environment. [cited 29 June 2023]. Available: https://cdphe.colorado.gov/animal-related-diseases/west-nile-virus/west-nile-virus-data

2. TIGER/Line Shapefile, Current, State, Colorado, County Subdivision. U.S. Department of Commerce, U.S. Census Bureau, Geography Division, Geospatial Products Branch (Point of Contact); Available: https://catalog.data.gov/dataset/tiger-line-shapefile-current-state-colorado-county-subdivision
